# EHMTI-0268. Long term efficacy of repetitive sphenopalatine blockade with bupivacaine vs. Saline with the tx360 device for treatment of chronic migraine

**DOI:** 10.1186/1129-2377-15-S1-E6

**Published:** 2014-09-18

**Authors:** RK Cady, HR Manley, RJ Cady, JG Tarrasch, AL Oh

**Affiliations:** 1 Clinvest, Banyan Group Inc., Springfield, USA

## Introduction

The sphenopalatine ganglion (SPG) resides within the pterygopalatine fossa in close proximity to the sphenopalatine foramen and has been implicated in orofacial pain conditions including migraine. Blocking the SPG using local anesthetics may relieve pain associated with chronic migraine.

## Aim

The purpose of this study was to evaluate safety and efficacy following treatment with repetitive SPG blocks using 0.5% bupivacaine vs. saline.

## Methods

This was an IRB approved, 2 center, randomized, double-blind, placebo controlled study with 41 consented adult subjects, meeting ICHD-II definition of chronic migraine. Subjects were randomized 2:1 following a 28 day baseline period and received either 0.3 mL of 0.5% bupivacaine (group A) or saline (group B) delivered to the mucosal surface of the SPG though each nares with the Tx360^®^ device. The procedure was repeated twice weekly for 6 weeks with follow-up visits 1 and 6 months post treatment.

## Results

There was a 6-day reduction in headache days for group A (p=.001) vs. a 2-day reduction for group B at 6 months (p=.09). HIT-6 scores were lower at 6 months vs. baseline for group A (p=.03) but not for Group B (p=.59). Furthermore, subjects receiving bupivacaine reported less work interference (p=.004), mood interference (p=.02), general activity (p=.05) relative to baseline. There were no significant changes in these measures for the saline group.

## Conclusion

These data suggest long-term benefits using repetitive SPG blockade with bupivacaine delivered by the Tx360^®^ device. Benefits include a reduction of headache days and improvement in several quality of life assessments.

Conflict of interest.

